# Effectiveness of Kenya's Community Health Strategy in delivering community-based maternal and newborn health care in Busia County, Kenya: non-randomized pre-test post test study

**Published:** 2012-12-26

**Authors:** Gilbert Wangalwa, Bennett Cudjoe, David Wamalwa, Yvonne Machira, Peter Ofware, Meshack Ndirangu, Festus Ilako

**Affiliations:** 1 AMREF in Kenya, Nairobi, Kenya; 2 AMREF in USA, New York, USA

**Keywords:** Child survival, neonatal care, maternal care, community-based care, community health strategy, AMREF, community health worker, Busia

## Abstract

**Background:**

Maternal mortality ratio and neonatal mortality rate trends in Kenya have remained unacceptably high in a decade. In 2007, the Ministry of Public Health and Sanitation adopted a community health strategy to reverse the poor health outcomes in order to meet Millennium Development Goals 4 and 5. It aims at strengthening community participation and its ability to take action towards health. The study aimed at evaluating the effectiveness of the strategy in improving maternal and neonatal health outcomes in Kenya.

**Methods:**

Between 2008 and 2010, the African Medical and Research Foundation implemented a community-based maternal and newborn care intervention package in Busia County using the community health strategy approach. An interventional, non-randomized pre-test post test study design was used to evaluate change in essential maternal and neonatal care practices among mothers with children aged 0 - 23 months.

**Results:**

There was statistically significant (p < 0.05) increase in attendance of at least four antenatal care visits (39% to 62%), deliveries by skilled birth attendants (31% to 57%), receiving intermittent preventive treatment (23% to 57%), testing for HIV during pregnancy (73% to 90%) and exclusive breastfeeding (20% to 52%).

**Conclusion:**

The significant increase in essential maternal and neonatal care practices demonstrates that, community health strategy is an appropriate platform to deliver community based interventions. The findings will be used by actors in the child survival community to improve current approaches, policies and practice in maternal and neonatal care.

## Background

Globally, the number of child deaths decreased from 12.5 million in 1990 to 8.8 million in 2008 [1]. However, neonatal deaths accounted for about one third of child deaths and are linked closely to slow progress in reduction of maternal mortality. It is globally estimated that about 342,900 maternal deaths occur each year [2]. There has been no substantial change in maternal mortality in sub-Saharan Africa over the past ten years and therefore progress towards MDGs 4 and 5 has remained slow in this region [3, 4]. The high maternal and newborn mortality in sub-Saharan Africa is related to unsafe maternal and newborn health (MNH) practices. Puerperal infections remain a major cause of maternal mortality, partly due to poorly observed rules of cleanliness and an unhygienic delivery environment. Most newborn deaths occur during the first week of life as a result of sepsis, birth asphyxia, birth injuries, complications of prematurity and low birth weight, and birth defects [4].

Continuum of care throughout pregnancy, childbirth and postnatal period is key in improving maternal and newborn health in order to reduce maternal and child morbidity and mortality [5]. However, promoting change in maternal and newborn health (MNH) behaviour is challenging due to knowledge barriers and service delivery gaps, traditional cultural beliefs and practices, lack of social support networks, financial constraints and inaccessibility of health units. Many women attend antenatal care (ANC) only once to get an ANC card, and they perceive traditional birth attendants (TBAs) to be effective care-givers. Maternal and neonatal danger signs are usually first treated with herbs, and women and caregivers only seek medical care when the condition worsens [6].

Experience over the past decade has shown that to improve maternal and newborn health and reduce morbidity and mortality, efforts should focus on building capacities at individual, family, and community levels to ensure appropriate self-care, prevention, and care-seeking behavior [7]. In limited resource settings, community-level interventions are potentially effective ways to address the problem at its roots, as decisions to seek and access health care are strongly influenced by the socio-cultural environment [8].

In northern India, the neonatal mortality rate (NMR) fell by 25% in two years [9] after community health workers (CHWs) were trained in essential newborn care, identification and special care of at-risk infants and referral to health facilities when appropriate. In Guatemala, the infant mortality rate declined by 85% when an immediate evidence-based treatment of infants began in the community, with accompanied referral to a nearby hospital [10]. A community-based trial conducted in Tanzania and Kenya demonstrated that, for areas in which maternal immunization against tetanus was not feasible, measures such as TBA training for safe and clean delivery and cord care were effective in decreasing perinatal, neonatal, and infant mortality [11].

The maternal and neonatal health trend in Kenya is a replica of other sub-Saharan African countries. The maternal mortality ratio (MMR) is estimated to be 488 women per 100,000 live births [12] which has not significantly changed over the last decade. Maternal deaths represent about 15% of all deaths of women aged 15-49 years. Between 2003 and 2008, the under-five and infant mortality declined by 36% and 32% respectively. However, neonatal mortality marginally declined by 6.1% [13].

In seeking to improve the health outcomes in Kenya, Kenya's Ministry of Public Health and Sanitation (MOPHS) through its National Health Sector Strategic Plan II (NHSSP II) emphasizes on promotion of individual and community health. The purpose of the NHSSP II is to strengthen health services through several strategies, one of which is the community health strategy. This is a community-based approach, through which households and communities take an active role in health and health-related development issues. Its goal is to enhance community access to health care by providing health care services for all cohorts and socio-economic groups at household and community levels; building the capacity of community health extension workers (CHEWs) and CHWs to provide community level services; strengthening health facility-community linkages; and raising the community's awareness of their rights to health services.

Despite widespread application of the community health strategy in Kenya since its inception in 2007, neither the Ministry of Public Health and Sanitation (MOPHS) nor its partners have developed empirical evidence to demonstrate the effectiveness of the strategy in improving maternal and neonatal care practices. The African Medical and Research Foundation (AMREF), a key partner with the MOPHS, resolved to evaluate the effectiveness of the community health strategy in delivering a community-based maternal and newborn care intervention in Butula and Funyula constituencies of Busia County, Kenya. The intervention's aim was to empower women, men, families, and communities to stay healthy, make healthy decisions and respond to obstetric and neonatal needs and emergencies; strengthen linkages between service delivery at levels one (community), two (dispensary), and three (health centre); and strengthen community [14]. The study objective was to evaluate the effectiveness of the community health strategy in delivering community-based maternal and newborn care intervention as a means of influencing adoption of essential maternal and newborn care (EMNC) practices among mothers with children aged 0-23 months. Our hypothesis was that consistent exposure of community and household members to essential maternal and newborn care messages through CHWs results in increased adoption of EMNC health practices by mothers or caretakers of newborns.

## Methods

### Study setting

The study was carried out in Butula and Funyula Constituencies of Busia County, Kenya, between October 2008 and June 2010. The two constituencies neighbor each other and border Uganda to the west and Lake Victoria to the south. They cover an area of 526.4 sq. kilometers, and have an estimated population of 215,384 persons, of whom approximately 50,000 are women of reproductive age (WRA) and 30,000 are children under five (CU5). The maternal mortality ratio for the two constituencies is estimated at 680 deaths per 100,000 live births [12]. The neonatal and infant mortality rates are estimated at 24 and 65 deaths per 1,000 live births respectively while under-five mortality rate is estimated at 121 deaths per 1000 live births. In both constituencies, more than 67% of people are classified as poor, with a mean monthly household income of US$42. The main causes of poverty include: lack of markets for produce, mainly fish and sugar cane; poor communication and transport infrastructure; and unreliable weather conditions. The population is predominantly Christian and of the Luhya ethnic group.

### The intervention

The community health strategy establishes a level one care unit (community unit) to serve a local population of 5,000 people. Each community unit has a cadre of well-trained CHWs who each provide services to 20 households. For every 25 CHWs there is one CHEW providing supervision and technical support. CHEWS are trained health personnel with certification in nursing or public health, and are MOPHS employees. Their responsibilities in the community health strategy include: facilitating trainings in the community, providing facilitative supervision to CHWs, and providing a link between CHWs and health facility [14].

The study team, through the District Health Management Teams (DHMTs) facilitated establishment of the CHS structures described above. A total of 45 community units (CUs) were established. Each unit recruited a Community Health Committee (CHC) with an average membership of 12 representatives elected by the community. Members were recruited in accordance with the Community Health Strategy guidelines and trained for three days using the CHC training curriculum. CHWs identification was also in accordance with the CHS guidelines which involved elections during village meetings convened by the respective village elders. The eligibility criteria for one to be a CHW was ability to read and write, permanent residence in the community and demonstrated commitment to the service of their neighbors. A total of 835 CHWs were elected (2 from each village) and trained for 7 days on community-based maternal and newborn care (CBMNC) intervention. Six hundred and eleven (611) were active or continuously provided level one services throughout the study period. As part of their responsibility to deliver maternal and newborn care services in the community, they made at least two household visits to women during pregnancy and three times after birth. During these visits, CHWs encouraged pregnant women to make at least four antenatal visits, conducted health screening for danger signs, provided health education, assisted in completing individual birth plans, advised on care of the newborn, monitored growth, and maintained records on services provided and maternal and neonatal health outcomes. The CHEWs provided continuous mentorship and supervision to CHWs [14]. The CHCs organized community dialogue sessions to raise awareness of maternal and newborn health issues with the aid of data displayed on a community chalk board. Deliberations of community dialogue days informed CHWs and CHEWs to plan during community action days for health service delivery in the community. The intervention was for a period of 22 months between October 2008 and June 2010.

### Study design, population and sampling

The study design was a pre-test and post-test non-randomized interventional study. The study population comprised of mothers with children aged 0 ≤ 23 months. A pre-determined sample size of 19 from each of seven strata (demarcated for effective supervision of the intervention) was used in the stratified technique to estimate the required sample size of women with children ≤ 23 months at each observation point (19x7=133). This translated to a sample size of 266 (133x2) at the two observation points. A sample of 133 gives a 95% confidence value or p-value < 5% [15]. Multiple stage sampling design was used to sample mothers with children ≤ 23 months to participate in the survey. Using villages with their respective number of households as a sample frame, 19 villages from each of the seven strata were randomly selected based on probability proportional to size. In each sampled village, a list of household heads provided the sampling frame and one household head was randomly sampled from the list to give a random start. Systematic sampling and parallel sampling were used to sample eligible respondents in the village. This process was followed in each of the sampled villages.

### Data collection and data quality control

A structured questionnaire was administered to eligible respondents through face-to-face interviews by trained research assistants. At each of the measurement time points, data collection was carried out for five days. Data collected included practices on antenatal care, malaria prevention and management, delivery services, thermal care, cord care, post-delivery care, breastfeeding and nutrition, integrated management of childhood illnesses, mother-to-child transmission of HIV.

Quality control measures put in place included selecting experienced research assistants and training them for three days, pre-testing the questionnaires, supervision of interviews by observing at least one interview per interviewer on each day, editing completed questionnaires in the field, and monitoring quality of data entry by verifying 10% of entered records.

### Data analysis

Inferential statistical analysis by use of Fisher's exact test was done to compute p-values with 95% confidence intervals (CIs) using SPSS software (version 16). Univariate analysis was done to determine the statistics for dependent variables that included: % of women with children aged 0 -23 months who attended at least 4 ANC visits; % of mothers with children aged 0-23 months whose children were delivered with assistance of a skilled health personnel;% of women with infants 0-5 months who attended postnatal care within two days after delivery; % of women who received at least two doses of SP for IPTp during ANC; % of infants age 0-5 months who were exclusively breastfed in the previous 24 hours; % of households with at least one ITN; % of women with children 0-23 months counseled and tested for HIV at ANC during their most recent pregnancy and knew their HIV status.

## Results

The mean age of respondents was 25.2 years (SD=5.6) and 25.9 years (SD=6.7) at pre-test and post-test respectively. Eighty-seven percent were married at pre-test as compared to eighty-five at post-test. Educational attainment was low, with 11% reporting having attained at least secondary level of education at pre-test and 19.5% at post-test. There was no significant difference in demographic characteristics of the respondents between the two observation points

There was a statistically significant (p ≤ 0.05) increase in essential maternal and neonatal care practices of four ANC visits, deliveries by skilled birth attendants, uptake of sulfadoxine pyrimethamine (SP) for intermittent preventive treatment (IPTp) for malaria in pregnancy, exclusive breastfeeding and knowledge of HIV status as shown in Table 1. However, the increase in postnatal check-up and ITN ownership was not statistically significant (p ≤ 0.05).

**Table 1:** Maternal and Neonatal Care Practices between Pre and Post Intervention Observation

Indicators	Pre-intervention (n=133)	Post-intervention (n=133)	Significance
At least 4 ANC visits made	39%	62%	p < 0.001
Deliveries by skilled health professionals	31%	57%	p < 0.001
Postnatal check-up within 2 days	52%	58%	p=0.36
Households with at least one ITN	89%	92%	P=0.68
Uptake two doses of SP for IPTp during ANC	23%	57%	p < 0.001
Pregnant women with knowledge of HIV status	73%	90%	p < 0.001
Exclusive breastfeeding	20%	52%	p < 0.001

## Discussion

The results show that community health strategy is an effective approach to delivering community-based intervention. This is evidenced by the significant changes in essential maternal and newborn care practices of ANC attendance, skilled deliveries and exclusive breastfeeding. The positive health outcomes documented by the study came about because household members had been empowered to make healthy decisions to respond to maternal and neonatal health needs. The strengthened linkages between the community and dispensaries and health centres enabled effective referrals from the community [7].

A continuum of care is needed throughout pregnancy, childbirth and the postnatal period in order to improve maternal and newborn health and reducing morbidity and mortality [5]. Efforts should focus on building capacities at individual and family levels [7]. This is confirmed by community based interventional studies in India and Guatemala where neonatal mortality rate and infant mortality reduced by 25% and 85% [9, 10] respectively. These positive changes facilitate behaviour change that improves pregnancy outcomes for women and health outcomes for newborns and infants. It also breaks the inherent traditional practices in future generations [1].

Most maternal and newborn recommended practices are acceptable to the community. However health system and community barriers are prevalent and need longer period to overcome. Women in rural settings prefer to deliver in health facilities but are constrained by the exorbitant delivery charges in such poverty stricken regions, absence of emergency transportation and inaccessible health units [16]. One-third of the pregnant women did not complete the recommended four ANC visits despite making one visit. They attend one ANC visit to secure an ANC card for use in case of emergencies [17]. Other barriers to completing four visits are lack of support from spouses.

The study had certain limitations that were not controlled for because of policy shifts during the study period. The effect of establishing new District Health Management Teams (DHMTs) for Funyula and Butula constituencies and the split of the Ministry of Health to the Ministry of Medical Services (MOMS) and the Ministry of Public Health and Sanitation (MOPHS) on health service delivery could not have been foreseen and were therefore not controlled for in the study. The study did not establish criteria to determine the weighted impact of number of household visits on dependent variables.

## Conclusion

The study shows that establishing community-based governance structures to organize and coordinate the activities of CHWs at community level, and linking the community with the formal health care system facilitated change in essential maternal and newborn care practices. However, some practices like postnatal care attendance did not change significantly. The implication on the Kenya health policy and practice is for the policy to focus on people centredness and participatory approaches in delivery of health care services. Further formative research is necessary to investigate barriers to postnatal care attendance.
